# Serum Copper-to-Zinc Ratio and Risk of Chronic Obstructive Pulmonary Disease: A Cohort Study

**DOI:** 10.1007/s00408-022-00591-6

**Published:** 2022-12-04

**Authors:** Setor K. Kunutsor, Ari Voutilainen, Jari A. Laukkanen

**Affiliations:** 1 Diabetes Research Centre, University of Leicester, Leicester General Hospital, Gwendolen Road, Leicester, UK; 2 National Institute for Health Research Bristol Biomedical Research Centre, University Hospitals Bristol and Weston NHS Foundation Trust and the, University of Bristol, Bristol, UK; 3 Musculoskeletal Research Unit, Translational Health Sciences, Bristol Medical School, University of Bristol, Learning & Research Building (Level 1), Southmead Hospital, Bristol, UK; 4 Institute of Public Health and Clinical Nutrition, University of Eastern Finland, Kuopio, Finland; 5 Institute of Clinical Medicine, Department of Medicine, University of Eastern Finland, Kuopio, Finland; 6 Department of Medicine, Central Finland Health Care District, Finland District, Jyväskylä, Finland

**Keywords:** Serum copper-to-zinc ratio, Serum copper, Serum zinc, Chronic obstructive pulmonary disease, Risk factor, Cohort study

## Abstract

**Purpose:**

Serum copper (Cu), zinc (Zn), and Cu/Zn-ratio have emerged as ageing-related biomarkers. We sought to assess the association between Cu/Zn-ratio and chronic obstructive pulmonary disease (COPD) risk.

**Methods:**

Serum Cu and Zn were measured using atomic absorption spectrometry in 2,503 men aged 42–61 years.

**Results:**

During a median follow-up of 27.1 years, 210 COPD cases occurred. Serum Cu/Zn-ratio and Cu concentrations were linearly associated with COPD risk, whereas the relationship was curvilinear for Zn and COPD risk. A unit increase in Cu/Zn-ratio was associated with an increased COPD risk in multivariable analysis (hazard ratio, HR 1.81; 95% CI 1.08–3.05). The corresponding adjusted HR (95% CI) was 3.17 (1.40–7.15) for Cu. Compared to the bottom tertile of Zn, the HRs (95% CIs) were 0.68 (0.48–0.97) and 1.01 (0.73–1.41) for the middle and top tertiles of Zn, respectively.

**Conclusions:**

Increased serum Cu/Zn-ratio and Cu concentrations were linearly associated with an increased COPD risk in men.

## Introduction

Chronic obstructive pulmonary disease (COPD) is an inflammatory respiratory disease that is associated with significant morbidity, mortality, and healthcare costs [1]. In 2019, there were 212.3 million global cases of COPD and it accounted for 3.3 million deaths, representing the third leading cause of death globally [1]. Major contributors to COPD include active smoking, comorbidities, genetics, occupational exposures, indoor and outdoor air pollution, and infections [2].

Though COPD is largely incurable once diagnosed, it is a preventable disease. Ageing also constitutes a major risk factor among the wide range of comorbidities and risk factors associated with COPD. This is due to the physiological changes associated with ageing such as weakening of the immune system [3], increased inflammation, and the high prevalence of comorbidities in older people. With global population ageing, there is increasing research focussed on identifying ageing-related biomarkers [4], which could be clinically useful for the prevention of ageing-related diseases such as COPD. Copper (Cu) and zinc (Zn) are essential micronutrients involved in several cellular processes [5, 6], and they have been identified as ageing-related biomarkers, given their close relationships with inflammatory parameters rather than the nutritional ones [7]. Insufficiency, deficiency, or toxic levels of these nutrients can lead to an increased incidence of age-related degenerative conditions such as vascular disease, cancer, and infections [8, 9]. Serum concentrations of Cu and Zn are biologically interrelated and strictly regulated by compensatory mechanisms that act to stabilize them within certain ranges of nutritional intake [10]. During pathological states such as systemic inflammation, serum Cu concentrations increase and that of Zn decreases [11]. Age-related chronic diseases are typically characterised by an increase in the concentrations of Cu-to-Zn ratio (Cu/Zn-ratio) [10]. There is documented observational cohort evidence on the relationships between elevated serum Cu/Zn-ratio and an increased risk of cardiovascular diseases [12], heart failure[13], cancer [12], all-cause mortality[7] as well as infectious diseases such as pneumonia [14]. Given the overall evidence, we hypothesized that serum Cu/Zn-ratio is linked to the risk of COPD. Thus, our aim was to assess the association between serum Cu/Zn-ratio and COPD risk, using a population-based prospective cohort of 2,503 middle-aged and older Finnish men. In a subsidiary analysis, we assessed the individual associations of serum Cu and Zn with COPD risk.

## Methods

Participants included in this study were part of the Kuopio Ischaemic Heart Disease Risk Factor Study (KIHD), a population-based prospective cohort study that comprised a representative sample of men aged 42–61 years recruited from Kuopio, eastern Finland. Baseline examinations were performed between March 1984 and December 1989. The study protocol was approved by the Research Ethics Committee of the University of Kuopio, and written informed consent was provided by each study participant. The study design, recruitment methods and assessment of risk markers have been described in detail in previous reports [13, 14]. **S**erum Cu and Zn concentrations were measured from frozen serum samples stored at − 20 ℃ for 1–5 years, using the PerkinElmer 306 atomic absorption spectrophotometer (Norwalk, Connecticut, USA). We included all incident cases of COPD that occurred from study enrolment through 2018. All KIHD study participants are under continuous annual surveillance for outcomes including COPD events using personal identification codes and no losses to follow-up have been recorded. Incident COPD cases were collected by data linkage to the National Hospital Discharge Register and a comprehensive review of hospital records. The diagnoses of COPD were made by qualified physicians based on clinical history, symptoms and spirometry findings [15]. Multivariable adjusted hazard ratios (HRs) with 95% confidence intervals (CIs) for incident COPD were estimated using Cox proportional hazard models. Stata version MP 17 (Stata Corp, College Station, Texas) was used to conduct all statistical analyses.

## Results

**Fig. 1:**
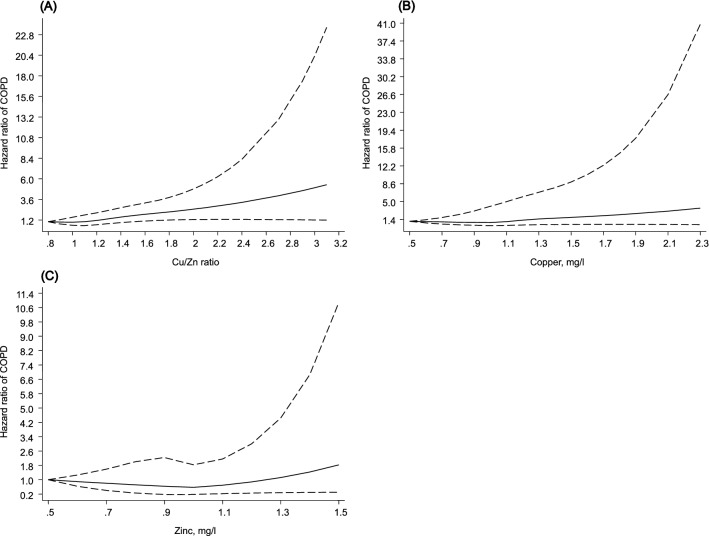
Restricted cubic splines of the hazard ratios of chronic obstructive pulmonary disease with serum Cu/Zn-ratio, Cu and Zn. **A** Serum Cu/Zn-ratio and COPD; **B** Serum Cu and COPD; **C** Serum Zn and COPD. Dashed lines represent the 95% confidence intervals for the spline model (solid line). Models were adjusted for age, body mass index, smoking status, history of type 2 diabetes, prevalent coronary heart disease, history of asthma, history of chronic bronchitis, history of tuberculosis, alcohol consumption, socioeconomic status, leisure-time physical activity, total energy intake, intake of fruits, berries and vegetables, and intake of processed and unprocessed red meat. *COPD* chronic obstructive pulmonary disease; *Cu* copper; *Zn* zinc

The overall mean (standard deviation, SD) age of study participants at recruitment was 53 (5) years. The means (SDs) of serum Cu/Zn-ratio, Cu, and Zn were 1.21 (0.27), 1.11 (0.18) and 0.94 (0.12), respectively (Table 1). During a median (interquartile range) follow-up of 27.1 (17.3–31.1) years, 210 COPD cases occurred. Multivariable restricted cubic spline curves suggested positive and linear relationships of serum Cu/Zn-ratio and Cu concentrations with COPD risk, whereas the relationship was inverse and curvilinear between serum Zn and COPD risk **(**Fig. 1). The HR (95% CI) for COPD per unit increase in serum Cu/Zn-ratio was 2.54 (1.60–4.04) in analysis adjusted for age, body mass index, smoking, history of type 2 diabetes, prevalent coronary heart disease, history of asthma, chronic bronchitis or tuberculosis, alcohol consumption, socioeconomic status, leisure-time physical activity, total energy intake, intake of fruits, berries and vegetables, and intake of processed and unprocessed red meat (model 2), which was attenuated to 1.81 (1.08–3.05) after further adjustment for high-sensitivity C-reactive protein (hsCRP), a potential mediator (Table 2). The corresponding adjusted HRs (95% CIs) were 1.79 (1.24–2.57) and 1.47 (1.00–2.16) comparing the top versus bottom tertiles of serum Cu/Zn-ratio. Higher concentrations of serum Cu were also associated with increased COPD risk but the corresponding adjusted HRs were more extreme than that those for serum Cu/Zn-ratio and COPD risk (Table 2). Compared to the bottom tertile of Zn, the HRs (95% CIs) for COPD were 0.66 (0.47–0.94) and 0.96 (0.69–1.34) for the middle and top tertiles of Zn, respectively, in analysis that adjusted for model 2 covariates (Table 2). The respective HRs (95% CIs) were 0.68 (0.48–0.97) and 1.01 (0.73–1.41) in further analysis adjusted for hsCRP.

**Table 1:** Baseline characteristics of study participants

Characteristics	Mean (SD) or median (IQR)
Serum copper-to-zinc ratio	1.21 (0.27)
Serum copper, mg/l	1.11 (0.18)
Serum zinc, mg/l	0.94 (0.12)
Questionnaire/prevalent conditions	
Age (years)	53 (5)
Alcohol consumption, g/week	31.8 (6.2–91.0)
History of type 2 diabetes, %	99 (4.0)
Current smoking, %	791 (31.6)
History of CHD, %	617 (24.7)
History of asthma, %	91 (3.6)
History of chronic bronchitis, %	189 (7.6)
History of tuberculosis, %	97 (3.9)
Physical measurements	
BMI, kg/m^2^	26.9 (3.6)
SBP, mmHg	134 (17)
DBP, mmHg	89 (11)
Physical activity, KJ/day	1204 (630–1999)
Socio-economic status	8.48 (4.23)
Blood-based markers	
Total cholesterol, mmol/l	5.90 (1.08)
HDL-C, mmol/l	1.29 (0.30)
Fasting plasma glucose, mmol/l	5.35 (1.28)
High-sensitivity C-reactive protein, mg/l	1.29 (0.71–2.48)
Dietary intakes	
Total energy intake, kJ/day	9855 (2595)
Processed and unprocessed red meat, g/day	145 (77)
Fruits, berries and vegetables, g/day	251 (156)

*BMI* body mass index; *CHD* coronary heart disease; *DBP* diastolic blood pressure; *GFR* glomerular filtration rate; *HDL-C* high-density lipoprotein cholesterol; *SD* standard deviation; *SBP* systolic blood pressure

**Table 2:** Associations of serum copper, zinc and copper-to-zinc ratio with risk of chronic obstructive pulmonary disease

Exposure	Events/ Total	Model 1		Model 2		Model 3	
		HR (95% CI)	*P* value	HR (95% CI)	*P* value	HR (95% CI)	*P* value
Serum copper-to-zinc ratio							
Per unit increase	210 / 2,503	4.51 (2.95–6.88)	< .001	2.54 (1.60–4.04)	< .001	1.81 (1.08–3.05)	.025
T1 (0.48–1.07)	46 / 835	ref		ref		ref	
T2 (1.08–1.27)	65 / 837	1.46 (1.00–2.13)	.051	1.20 (0.82–1.76)	.34	1.11 (0.76–1.64)	.59
T3 (1.28–3.12)	99 / 831	2.59 (1.82–3.68)	< .001	1.79 (1.24–2.57)	.002	1.47 (1.00–2.16)	.052
Serum copper, mg/l							
Per unit increase	210 / 2,503	10.33 (5.28–20.20)	< .001	5.11 (2.49–10.47)	< .001	3.17 (1.40–7.15)	.005
T1 (0.46–1.02)	42 / 875	ref		ref		ref	
T2 (1.03–1.17)	73 / 826	1.94 (1.33–2.83)	.001	1.79 (1.22–2.63)	.003	1.65 (1.12–2.43)	.012
T3 (1.18–2.32)	95 / 802	3.10 (2.16–4.46)	< .001	2.17 (1.49–3.15)	< .001	1.79 (1.20–2.66)	.004
Serum zinc, mg/l							
T1 (0.50–0.89)	94 / 911	ref		ref		ref	
T2 (0.90–0.98)	52 / 802	0.57 (0.41–0.80)	.001	0.66 (0.47–0.94)	.021	0.68 (0.48–0.97)	.033
T3 (0.99–1.62)	64 / 790	0.78 (0.56–1.07)	.12	0.96 (0.69–1.34)	.81	1.01 (0.73–1.41)	.93

Model 1: Adjusted for age. Model 2: Model 1 plus body mass index, smoking status, history of type 2 diabetes, prevalent coronary heart disease, history of asthma, history of chronic bronchitis, history of tuberculosis, alcohol consumption, socioeconomic status, leisure-time physical activity, total energy intake, intake of fruits, berries and vegetables, and intake of processed and unprocessed red meat. Model 3: Model 2 plus high-sensitivity C-reactive protein

*CI* confidence interval; *CRF* cardiorespiratory fitness; *HR* hazard ratio; *ref* reference; *T* tertile

## Discussion

In a cohort of middle-aged and older Finnish men, higher serum Cu/Zn-ratio was associated with an increased risk of COPD in a linear dose–response manner. In separate evaluations of serum Cu and Zn, increased serum Cu was associated with increased COPD risk in a graded manner, whereas serum Zn was inversely associated with COPD risk in a curvilinear manner. On adjustment for inflammation (as measured by hsCRP), the associations were markedly attenuated but remained significant.

These findings reflect the fact that inflammatory pathways are involved in the development of COPD. High serum Cu concentrations increase COPD risk via increased inflammation, given its close link with ceruloplasmin, which is an acute phase response protein and markedly increased during inflammation [16]. Given that the associations persisted following further adjustment for inflammation, other mechanistic pathways may underline the observed associations of serum Cu/Zn-ratio and Cu and Zn concentrations with the risk of COPD. Despite its beneficial role in numerous biological processes, serum Cu can exhibit toxic effects in high concentrations. Increased serum Cu concentrations may also increase COPD risk via oxidative stress and activation of lung fibroblasts, which lead to pulmonary fibrosis [17]. Zinc has antagonistic effects on the toxicity of Cu [18] and may play a protective role in the development of COPD via its anti-inflammatory, antioxidant, immune, and metabolic modulatory properties [19].

The overall findings suggest that serum Cu/Zn-ratio, Cu and Zn could be potential risk markers for incident COPD. Therefore, measurement of serum Cu and Zn concentrations as well as Cu/Zn-ratio could be used to identify individuals at high risk of COPD. It is well known that Zn deficiency in old age is usually due to insufficient dietary Zn intake, reduced intestinal absorption or increased losses [20], hence, micronutrient-based therapies that can boost Zn levels could also provide optimal concentrations of serum Cu/Zn-ratio and reduce the risk of COPD. Zinc supplementation has been observed to have favourable effects on oxidant–antioxidant balance in patients with COPD [21].

This is the first prospective evaluation of the associations of serum Cu/Zn-ratio, Cu and Zn with the risk of COPD. Other strengths include the use of a representative sample of Finnish middle-aged to older men, use of a relatively large prospective cohort with long-term follow-up, the zero loss to follow-up, availability of a comprehensive panel of essential covariates which enabled adequate control of confounding factors, and assessment of the dose–response relationships. The limitations include the possibility of selection bias, the generalisability of the findings to only middle-aged and older men and biases inherent in observational cohort designs such as reverse causation and residual confounding due to errors in measured confounders and/or relevant unmeasured confounders such as environmental factors and comorbidities that affect values of the exposures. Furthermore, we did not have data on changes in nutritional status and biomarkers over the long-term follow-up.

In conclusion, increased concentrations of serum Cu/Zn-ratio and Cu were associated with an increased risk of incident COPD in middle-aged and older Finnish men, consistent with linear dose–response relationships. The relationship between serum Zn and COPD was inverse and nonlinear. Furthermore, the associations were independent of several lifestyle and dietary variables as well as hsCRP. Given this is the first report to demonstrate these associations, other large-scale studies are needed to confirm these findings.

## Abbreviations

CI: Confidence interval
COPD: Chronic obstructive pulmonary disease
Cu: Copper
HR: Hazard ratio
hsCRP: High-sensitivity C-reactive protein
IQR: Interquartile range
KIHD: Kuopio ischaemic heart Disease
SD: Standard deviation
Zn: Zinc
